# Risk of Hepatitis B transmission by healthcare workers – a systematic review 

**DOI:** 10.3205/dgkh000572

**Published:** 2025-08-15

**Authors:** Roland Diel, Albert Nienhaus

**Affiliations:** 1 Institute of Epidemiology, University Medical Hospital Schleswig-Holstein, Kiel, Germany; 2 Institution for Statutory Accident Insurance and Prevention in the Health and Welfare Services (BGW), Hamburg, Germany

**Keywords:** health care workers, hepatitis B, transmission, infectivity, professional-to-patient, guidelines, exposure prone procedures

## Abstract

**Background:**

The risk of transmission of hepatitis B virus (HBV) to healthcare workers (HCW) is well known. However, evidence for supporting guidelines with respect to exclusion of infected HCW from exposure prone procedures (EPP) remains poorly characterized.

**Method:**

A systematic review of studies published providing serological data for transmission of HBV infected HCW to patients was performed. Following preferred reporting items for systematic reviews and meta-analyses (PRISMA) we searched MEDLINE, Scopus and Cochrane databases to identify publications prior to September 2024.

**Results:**

The literature search yielded 311 studies and 39 from nine countries met the inclusion criteria. A total 53 of HCW were considered as source cases of transmission and 25,000 individuals tested for at least one HBV marker. 66 transmissions by HCW to patients were confirmed through DNA analysis; in 100 patients HBV transmissions were considered probable and in 480 patients at least possible. Of the 36 studies in which HBeAg in HCW was determined, the antigen was positive in 29 studies (80.6%), and negative only in seven studies (19.4%), comprising a total of only 31 and 17 HCW, respectively. The HBV viral load of the transmitting HCW was conducted in only 8 studies including 18 HCW, of those four were HBeAg-positive and 14 HBeAg-negative. Although the viral load in HBeAg-negative sources generally was 10 times lower than in HBeAg-positives, considerable variability was seen in HBeAg-negatives with overlapping values up to 1.5×10^9^ copies/mL. A HBV DNA value of 4×10^4^ copies/mL represents the lower threshold for transmissibility for 18 source cases in all studies, however, for the other 35 no measurements were available. Due to the low evidence on defining an HBV DNA viral load below which HBV transmission from HCW to patients appears unlikely, the safety thresholds for excluding infected HCW from performing EPP in most recent national guidelines (UK, Germany, the Netherlands and the US) still differ by factors of as much as 5 (200 IU/mL to 1,000 IU/mL).

**Conclusions:**

The published literature on HBV transmission from HCW to patients is sparse and offers only limited guidance on national prevention guidelines.

## Zusammenfassung

**Hintergrund:**

Das Risiko der Übertragung des Hepatitis B-Virus (HBV) durch medizinisches Personal (MP) auf Patienten ist hinreichend dokumentiert. Allerdings ist die Evidenz zur Unterstützung von Richtlinien bezüglich des Ausschlusses infizierter Beschäftigter von invasiven Tätigkeiten mit Übertragungsgefahr („exposed prone procedures“, EPP) bislang unzureichend charakterisiert.

**Methode:**

Es wurde eine systematische Übersichtsarbeit zu Studien mit serologischen Daten zur Übertragung von HBV durch infiziertes MP auf Patienten durchgeführt. Entsprechend der PRISMA-Kriterien wurden die Datenbanken MEDLINE, Scopus und Cochrane bis September 2024 durchsucht.

**Ergebnisse:**

Die Literatursuche ergab 311 Studien, von denen 39 aus neun Ländern die Einschlusskriterien erfüllten. Insgesamt wurden 53 Beschäftigte als mögliche Übertragungsquelle dokumentiert und 25.000 Personen auf mindestens einen HBV-Marker getestet. 66 Übertragungen durch MP wurden mittels DNA-Analyse bestätigt; bei 100 Patienten galt eine HBV-Übertragung als wahrscheinlich und bei 480 Patienten als zumindest möglich. In 36 Studien wurde das HBe-Antigen (HBeAg) des MP bestimmt; in 29 Studien (80,6%) war es positiv und in nur sieben Studien (19,4%) negativ, wobei beide Gruppen nur 31 bzw. 17 Beschäftigte umfassten. Die HBV-Viruslast des übertragenden MP wurde in nur 8 Studien mit insgesamt 18 Beschäftigten gemessen. Von diesen waren vier HBeAg-positiv und 14 HBeAg-negativ. Obwohl die Viruslast bei HBeAg-negativen Fällen im Durchschnitt um den Faktor 10 niedriger war als bei HBeAg-positiven, zeigte sich auch bei HBeAg-Negativen eine erhebliche Variabilität mit überlappenden Werten von bis zu 1,5×10^9^ Kopien/ml. Ein HBV-DNA-Wert von 4×10^4^ Kopien/ml stellte die untere Schwelle für die Übertragbarkeit bei 18 Fällen in allen Studien dar; für die übrigen 35 Fälle lagen jedoch keine Messwerte vor.

Aufgrund der geringen Evidenz zur Bestimmung einer HBV-DNA-Viruslast, unterhalb derer eine Übertragung von MP auf Patienten unwahrscheinlich erscheint, unterscheiden sich die Sicherheitsschwellen für den Ausschluss infizierter Beschäftigter von EPP in den aktuellen nationalen Leitlinien (Großbritannien, Deutschland, den Niederlanden und den USA) um den Faktor 5 (200 IU/mL bis 1.000 IU/mL).

**Schlussfolgerungen:**

Die veröffentlichte Literatur zur HBV-Übertragung von MP auf Patienten ist begrenzt und bietet nur eine eingeschränkte Orientierung zur Erstellung nationaler Präventions-richtlinien.

## Introduction

The risk of occupational transmission hepatitis B virus (HBV) from healthcare workers (HCW) to patients is well established. The primary route of infection from HCW who perform exposure prone procedures (EPP) on patients is the percutaneous (needlestick and other sharps injuries), followed in prominence by the mucocutaneous [1], [2]. In Germany, hepatitis B vaccination has been recommended since 1982 for individuals at elevated risk of infection, such as HCW. Much later, in 1995, a recommendation followed that all infants and children be vaccinated against HBV [3]. However, a 2022 study by the German Robert Koch Institute found no significant difference in rates of past HBV infection, as defined by antibodies to hepatitis B core antigen (anti-HBc), between HCW and other occupational groups, with the overall prevalence at 5.3% for men and 4.8% for women [4]. Since 2000, when there were 227 healthcare-related HBV cases [5], the annual number of cases reported to the Institution for Statutory Accident Insurance and Prevention in the Health and Welfare Services (BGW), which insures approximately 40% of employees in the German medical sector, has indeed decreased since then. Nevertheless, in recent years the annual number of HCW suspected of occupationally acquired HBV infection have hovered around 15–30 [6] whereby the total number of chronic HBsAg carriers in HCW remains unknown. 

Although the overall risk of HBV transmission from infected HCW to patients in our developed, low-prevalence nation can be considered low, minimizing this risk to patients is an uncontested ethical duty for all HCW. Hence the keeping of robust guidance in place remains a priority. One of the first published set of recommendations for the prevention of nosocomial HBV transmission to patients in the U.S. was that of Lettau et al. 1986 [7]. Since then, numerous guidelines for managing infected HCW have been published in high-income countries. These guidelines have been continuously updated in response to a gradually emerging number of published studies on HBV transmission from “professionals” to patients, which have often been summarized in mini-reviews (e.g. [8], [9], [10]). Beyond that, only Lewis et al. [11] have presented a more comprehensive overview, in 2015. Surprisingly, to date there has been no systematic review that deals with the question of placing restrictions on the types of medical activities that may be undertaken by personnel infected with HBV. What evidence exists to justify such restrictions, and what is standing in the way of a decision-making process on the matter? Sensing a need, we have now identified and critically analysed the studies available on HBV transmission to patients in healthcare settings, with particular interest directed at the impact they have had on national guidelines. 

## Methods

### Definition of HCW

HCW were defined as all medical, dental, nursing, obstetric or assisting persons in different areas, e.g. hospitals, outpatient clinics, doctors’ surgeries practices, dialysis facilities, nursing homes and out-patient care facilities. The decisive factor in the above-mentioned activities was the existence of a plausible transmission path.

### Study selection 

We searched the literature published before August 1, 2024 using PubMed, Cochrane and Scopus databases. The following terms were used in Boolean searching: “hepatitis B,” “transmission,” “health care workers” “healthcare workers,” “professional-to-patient” and “professional to patient”. Only studies written in English language published that provided original serological data on suggested nosocomial HBV transmission to patients were considered, without restriction to publication date. 

Review articles, guidelines, conference abstracts, commentaries, editorials, articles including no serological HBV markers at all and articles with a central theme diverging from or not related to reported professional-to-patient transmission of HBV were excluded. No restrictions were made regarding study design, patient subpopulation, or data collection (prospective or retrospective). If there were studies reporting duplicate data, the study with the most up-to-date and complete data was used. Reference lists of the included articles as well as of the review articles were manually screened to check for additional relevant articles. All records were transferred into the EndNote reference manager, which automatically removed duplicates. The preferred reporting items for systematic reviews and meta-analysis (PRISMA) standards 2020 guidelines were followed [12]. 

### Data extraction 

Relevant studies were independently selected by two reviewing authors (RD and AN), who screened each article title and abstract initially, and then went on to review an article’s full text as required. Any discrepancies were resolved by consensus. The following variables were recorded, if available:

1. country of study and year of publication;
2. study period;
3. type of study;
4. occupation or working environment of the suspected carrier;
5. number of persons tested (including staff members if applicable and secondary HBV cases identified);
6. HBeAg status (positive, negative or not done) and HBsAg subtype (if available);
7. HBV viral load (number of HBV copies per mL, if available);
8. number of transmissions (separated by confirmed, probable or possible transmission);
9. suspected route of transmission. Depending on the publications’ preferences, HBV viremia levels were expressed in HBV DNA copies/mL or IU/mL, where five HBV DNA copies correspond to approximately one IU of HBV DNA.

Where the VERSANT HBV DNA 3.0 Assay was used, a conversion factor of 5.26 HBV DNA copies per IU was applied as published in Ronsin et al. [13].

### Definition of transmission probability 

HBV transmission was considered confirmed when, beyond the obvious strong epidemiological link, genetic sequencing of the HBV strain from the source and recipient showed an identical or highly related viral genome. Probable transmissions were defined as cases where genetic sequencing was not done or was inconclusive, but the subtype of HBV infecting both the HCW and patient were identical. HBV transmissions were considered possible when epidemiologic links were established (e.g., consistency with the timing of surgery), infected patients had no other plausible risk factors for HBV acquisition but virologic subtyping data was not available. 

### Assessment of study quality 

All of the studies included under these criteria were “look-back” studies to identify either the source of HBV infection or – vice versa – to find secondary cases of transmission. Since test results were highly dependent on the availability of patient records, the chosen observation period, laboratory capacity, and ultimately the willingness to undergo serological testing, most collected data carried the risk of either selection bias and/or or information bias. Therefore, a formal assessment of study quality, e.g. by using the Joanna Briggs Institute (JBI) critical appraisal of prevalence studies scale [14], [15] was not considered appropriate. 

## Results

### Study availability 

**Figure 1:**
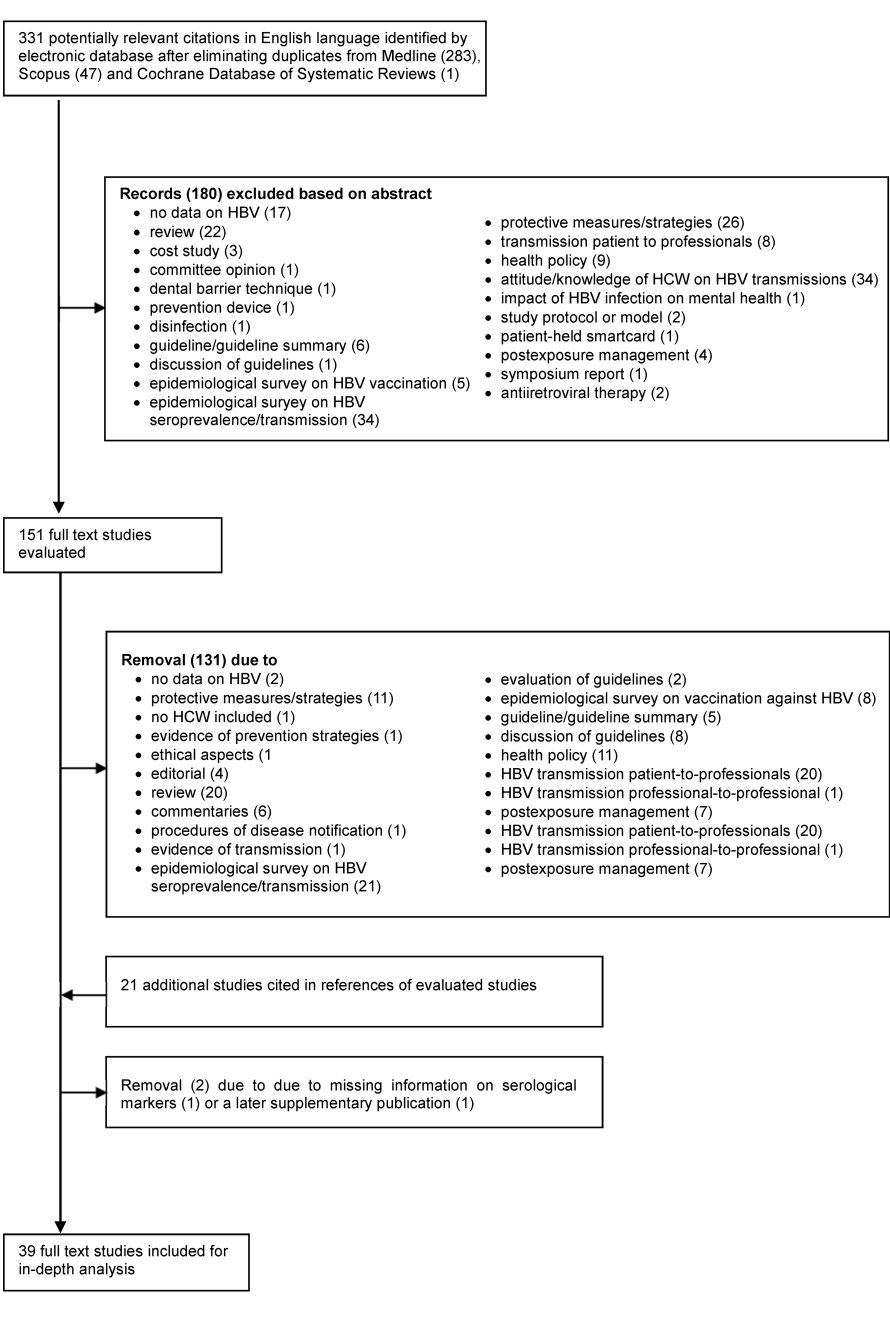
PRISMA flow diagram of study selection

Figure 1 (Fig. 1) presents a flow diagram of the literature search results. In the selection process, 331 journal abstracts in English (283 articles in PubMed, 79 in Scopus and 1 in the Cochrane review database) were identified. After 180 records were excluded based on their abstracts, a total of 151 abstracts were read in full text. Of those, 20 studies were eligible for inclusion. They were supplemented by 21 studies that were not covered by the terms of our search strategy and only found as references in other studies which had been read in full text. Three further studies could not be considered: the full text of Goodwin’s 1975 study [16] was no longer available. In Garibaldi’s study, [17] the nurse suspected of HBV transmission had not been tested for HBsAg, and testing following her recovery identified neither antigen from nor antibody to the virus. Furthermore, Grob’s 1975 letter [18] was replaced by an extended version of his findings published in 1981. Finally, 39 studies published in peer-review journals [19], [20], [21], [22], [23], [24], [25], [26], [27], [28], [29], [30], [31], [32], [33], [34], [35], [36], [37], [38], [39], [40], [41], [42], [43], [44], [45], [46], [47], [48], [49], [50], [51], [52], [53], [54], [55], [56], [57], [58] were included for in-depth analysis. Two reports by Martyn Halle [42], [43], published almost simultaneously in the BMJ and complementing each other in content, were regarded as a single unit. 

### Study characteristics 

The characteristics of the included studies are presented in Attachment 1. The studies came from nine high-income countries and cover a publication period from 1974 to 2013. Most were from the UK (18/39, or 46.2%), the USA (13/39, or 33.3%), Canada and the Netherlands (each 2/39, or 5.1%). Further source countries were France, Japan, Norway and Switzerland (each 1, or 2.6%). 

14 of the 39 studies (35.9%) were retrospective cohort studies, 11 studies had a cross-sectional design (28.2%), while seven studies were case-series or case reports, respectively. Overall, four case-control studies were included, and three studies found the sources of transmission by case-contact tracing. A total of 25,000 individuals (depending on the study design, possibly including control patients) underwent serological testing for at least one HBV marker or had corresponding results already available. In this context, 66 cases of transmission by HCW to patients were confirmed through DNA analysis; in 100 patients HBV transmissions were considered probable, and in 480 patients they were at least possible after excluding other reasonable risk factors. The sample size of the studies ranged from 1 to 9,620 participants.

In seven studies, HBV was transmitted by dentists or oral surgeons; in one study, transmission occurred through acupuncture, in another through an electroencephalogram technician, and in one study through a general practitioner. In the other 29 studies, the transmissions were caused by surgeons from various specialties outside the dental field or cardiopulmonary surgical support personnel. Only in 9 studies were specific hand injuries of the transmitting HCW documented (beyond the mere absence of gloves or double-gloving). Surprisingly, in 24 studies, no obvious mode of transmission was identified at all.

### Serological HBV markers and viral load 

In three studies [19], [28], [54], the presence of HBeAg in the HCW suspected to be the source of transmission was not determined; in two studies [27], [32], the determination was missing in a subgroup of patients. The vast majority of cases involved transmission from an HBeAg-positive HCW: Of the 36 studies examining HBeAg in HCW, 29 (80.6%) demonstrated positive results in 31 HCW. Conversely, 7 studies (19.4%) showed negative results in 17 HCW.

HBV DNA load was determined in only 18 infected HCW. Unfortunately, only for four of the 31 HBeAg-positive HCW was the HBV viral load available; it was in all four cases higher than or nearly 10^8^ copies/mL (see Attachment 1). No viral load values could be obtained for the remaining 27 HBeAG-positive cases. Of interest, within Corden’s laboratory-based study [53], a different group of 31 HCW HBeAg carriers, not suspected sources in transmission events, showed high levels of HBV DNA (median 8.2 lg 10 copies/mL, i.e. equivalent to approximately 1.58×10^8^ HBV DNA copies/mL serum), suggesting significant viral replication in HbeAg positives.

In 12 of the total of 14 HBeAg-negative HCW in whom the viral load was measured, HBV DNA copies were found to be at least one lg factor lower than the 10^8^ limit, with values ranging between 4×10^4^ copies/mL and 1×10^7^ (see Attachment 1). That first value is the lowest viral load reported among HCW infecting patients in the 39 studies. However, in two HBeAg-negative surgeons in Corden’s study [53] who had transmitted HBV, values which would have been expected in HBeAg positives (6.3×10^8^ and 1.5×10^9^ copies/mL) were measured. Indeed, in this study, the total of 136 HBeAg-negative carriers in whom HBV DNA could be detected and quantified had a median level of only 3.6 lg 10 copies/mL (equivalent to approximately 4,000 copies/mL), suggesting that pronounced viremia in HBeAg-negative carriers is rare. Nevertheless, the broad range of viral load of 5.7 lg copies (equivalent to approximately 5.0×10^5^ copies/mL) within this group indicates considerable variability. Thus, molecular tests more accurately reflect infectivity than does HBeAg status. 

## Discussion

### Risk assessment 

To our knowledge, this analysis is the first systematic review examining published reports of HBV transmission from HCW to patients. Across 39 studies with serological test results spanning the 36 years from 1974 to 2010, only 66 confirmed and 414 probable or possible cases of transmission were identified, and the viral load was determined in only 18 HCW in eight studies. It follows that the published data can only be of limited value in developing national recommendations for placing restrictions, or not, on the continued employment of HBV-infected HCW. Nevertheless, they provide important guidance.

Firstly, in the overwhelming majority (82.9%) of the 35 studies, in which HBeAg was used to investigate cases of nosocomial HBV transmission, antigen-positive caregivers were identified. HBeAg-positivity therefore places a HCW at especially high risk of transmitting the virus to patients.

Secondly, the results suggest that the management of HBV-infected HCWs cannot not be based on the presence of HBeAg HBV DNA level appears to be a much more useful tool. Cohen’s study shows that even HBeAG-negative HCW may have high levels of HBV DNA. Although the subjects had no documented HBV transmission on their record, it may be considered that such events be expected. Excluding HCW from performing EPP based solely on the presence of HBeAg as it has been suggested in previous decades (e.g. [59]) may not be sufficient. 

Thirdly, and importantly, only one HCW involved in HBV transmission to patients showed a viral load below 10^5^ HBV DNA copies/mL (20,000 IU/mL) – specifically, 4×10^4^ HBV DNA copies/mL [53]. Nevertheless, as in our review the viral load was only available in 18 out of the total of 53 HCW who were suggested or confirmed as source of transmission in the 39 investigated studies, there is a lack of evidence on the actual number of HBV copies/mL associated with transmission across the studies. Furthermore, the mostly one-time measured HBV DNA is merely a snapshot of the viral load and does not take into account spontaneous fluctuations in an individual’s viral load over time, as learned from other studies, e.g. [60], [61]. In Tedder’s study [61] sampling of 20 carriers whose sera contained anti-HBe over a mean of 5.3 years found – compared with initial values – an increase in HBV DNA by an average of 0.89 logarithmic units (base 10), corresponding to approximately a 7.8-fold rise, during the observation period.

Thus, it seems necessary to ensure a safety margin, by establishing a viral load level above which EPP should be restricted. In this way, one may maximize the professional utility of affected HCWs while ensuring a great degree of patient protection.

### Translation of the results of HCW transmission studies into national guidelines 

It goes without saying that all HCW for whom HBV vaccination is contraindicated, who decline vaccination, or who are non-responders to vaccination (i.e. anti-HBs <10 IU/L), should be investigated for persistent HBV infection, i.e. tested for presence of HBsAg or anti-HBc in the absence of HBsAg. Individuals testing positive must endure regular HBV DNA level monitoring. Historically, the recommendations of individual countries have varied significantly, both in terms of the exclusion of HBeAg-negative HCW and the threshold for HBV DNA levels used in decision-making. However, a considerable alignment can now be observed. 

The European Consensus group guidelines published in 2003 agreed that each country should individually determine the HBV DNA level cut off on its own, but recommends general exclusion of HBeAg-positive HCW from performing EPP [62]. For HBeAg-negative HCW it sets the threshold for performing EPP at 10^4^ HBV DNA copies/mL, equivalent to 2,000 IU/mL, declaring that this cut-off provides a balance between risk of transmission and loss of specialist HCWs. This cut off would also take into any account sudden rises of HBV DNA levels seen due to natural fluctuations or the potential emergence of resistant virus during lamivudine monotherapy. 

In Germany’s 2020 DVV recommendations [63] and in the S3 guidelines of the German Society for Gastroenterology, Digestive and Metabolic Diseases (DGVS) on the prophylaxis, diagnosis, and treatment of Hepatitis B virus infection [64], HBV DNA concentrations exceeding 10^5^ copies/mL (20,000 IU/mL) are classified as incompatible with EPP. On the other hand, HBV- infected HCW who are HBeAg-negative and whose viral loads do not exceed 10^3^ copies/mL (200 IU/mL) need not be restricted from performing EPP or from any other areas of work. However, even these HCW should have their viral loads tested regularly at 12 monthly intervals. Mathematically, this approach introduces a safety margin of 100-fold compared to the “usual” 10^5^ HBV DNA transmission limit. Specifically, the German recommendations introduce an intermediate category for a viral load between 200 and 20,000 IU/mL where EPP should only be performed in exceptional cases, following individual case assessment by a commission and under increased safety precautions (e.g., double gloves with a needle-stick indicator). Alternatively, restrictions should be imposed, limiting the individual to endoscopic or laparoscopic procedures.

These recommendations are in line with the current UK guidelines, the alignment of which with the current German recommendations, however, has only occurred gradually. In the 2,000 UK guidelines [65], all HBeAg-positive HCWs were excluded from EPP with distinct risk of bleed-back. In all other cases, no restrictions on professional activity were considered necessary for HCW, if HBV DNA concentrations were basically below 1,000 copies/mL (200 IU/mL). In light of the availability of 3^rd^ generation nucleoside analogues, the updated UK guidance of 2007 [66] lifted this apodictic cut-off a bit. While still excluding HBeAg-positive HCW, the new guidelines allowed HBeAg-negative HCW on continuous antiviral treatment to perform EPP, even when their pre-treatment HBV DNA levels were between 10^3^ and 10^5^ copies/mL. This was provided their HBV DNA levels were now suppressed to below 10^3^ copies/mL and retesting was done every 3 months. It was only an update of the UK Advisory Panel for HCW Infected with Bloodborne Viruses (UKAP) in 2020 that removed past barriers, restricting HCW from performing EPPs solely on the basis of HBV DNA level, regardless of HBeAg and/or treatment status. In the latest edition dated April 2024, a case-by-case approach based on clinical judgment was recommended for HBV DNA levels between 60 and below 200 IU/mL to decide whether no action was considered necessary or whether a second test should be done 10 days later to verify that the viral load remains below the threshold [67].

The first recommendations of the Netherlands had focused on the results of Corden’s study: In their 3^rd^ edition of 2012 [68] the guideline commission decided to maintain the threshold of 10^5^ HBV-DNA copies/mL, or 20,000 IU/mL, based on the consideration that HBV transmission at a viral load below 10^5^ HBVDNA copies/mL would be a rare exception. Lowering the threshold to 10³ HBV-DNA copies/mL (as chosen in the UK guidelines at that time due to the natural fluctuation of viral load over time) would result in only a minimal reduction in patient risk while disproportionately increasing the number of affected healthcare workers who would be entirely or partially excluded from their profession.

In the latest (4^th^) edition, published in 2021 [69], however, these recommendation were changed, now excluding Individuals with an HBV DNA level exceeding 1,000 IU/mL, or 5,000 copies/ml. As of March 2020, all HBV-infected HCW monitored by the Netherlands Commission – whether on antiviral treatment or not – showed significantly lower viremia levels than the previous threshold of 20,000 IU/mL. This is attributable to effective viraemic control achieved with HBV antiviral therapies, notably tenofovir and entecavir.

The latest guidance (2020) from the Society for Healthcare Epidemiology of America (SHEA) [70] also restricts HCWs with HBV from performing EPP solely on the basis of HBV DNA level (independent of antiviral treatment or HBeAg status) and now adopts a cut-off level of equal to or higher than 1,000 IU/mL instead of 10^4^ copies/mL recommended in the version issued 10 years earlier [71]. This is now in line with the older 2012 CDC recommendations, which had already considered a threshold value of <1,000 IU/mL serum HBV DNA "safe" for practice [72]. 

## Conclusions

Limited published research on HBV transmission from HCW to patients leaves the occupational health community without a definitive viral load threshold for negligible risk. This scarcity of evidence has historically led to disparate national prevention guidelines, with thresholds ranging from 200 to 20,000 IU/mL. However, a recent convergence towards lower thresholds improves patient safety, the paramount concern of public health guidance.

## Notes

### Competing interests

The authors declare that they have no competing interests.

### Author’s ORCIDs

- Diel R: https://orcid.org/0000-0001-8304-7709
- Nienhaus A: https://orcid.org/0000-0003-1881-7302

## Supplementary Material

Results of suggested HBV transmission by HCW
