# Supplementary material for: Risk of Hepatitis B transmission by healthcare workers – a systematic review
Source: GMS Hyg Infect Control. 2025 Aug 15;20:Doc43. doi: 10.3205/dgkh000572 (PMC12447767; doi:10.3205/dgkh000572)
Supplement: Results of suggested HBV transmission by HCW [file HIC-20-43-s-001.pdf]

Attachment

Results of suggested HBV transmission by HCW

Author, country, publication year	Study period	Study type	Occupation	Persons tested (no.)	Assessment of HBeAg, HBsAg subtype	HBV DNA (copies/mL)	Degree of confirmation	Likely mode of transmission
Levin et al., USA, 1974 [19]	1972–1973	Case series	Dentist in private practice	21 (13 patients, 8 staff members)	Not done	Not done	13 (possible)	Small abrasions or cuts on the dentist´s hands
Snydman et al., USA, 1976 [20]	1974–1975	Case-control	Respiratory therapist (open heart surgery)	45 (17 patients with open-heart surgery)	Positive, subtype ayw	Not done	4 (4 probable)	HCW did not wear gloves, had an exudative dermatitis on hands, and reused syringes
Rimland et al., USA, 1977 [21]	1971–1974	Retrospective cohort	Oral surgeon in private practice	42	Positive, subtype ay	Not done	32 (10 probable, 22 possible)	HCW did not wear gloves and reported sustaining hand cuts
PHLS, UK, 1980 [22]	Jan–Jul 1978	Case-control	Surgical registrar, gynaecologic surgery	1,332 (589 patients and 743 controls with “known outcome”)	Positive, subtype ad	Not done	8 (6 probable, 2 only possible as in those patient HBsAg titre was too low for sub-typing)	None
Grob et al., Switzerland, 1981 [23]	1973–1979	Cross-sectional	General practitioner	150	Positive, no subtyping	Not done	41 (41 possible)	Wearing no gloves the practitioner sometimes had small lesions on his fingers
Hadler et al., USA, 1981 [24]	1978 (Apr–Sep)	Cross sectional (with nested case-control design)	Dentist in private practice	764	Positive, subtype adw	Not done	6 (2 probable, 4 possible[a])	HCW did not wear gloves
Haerem et al., Norway, 1981 [25]	1978 (1st Jan–Jun 30)	Retrospective cohort	Cardiac surgeon	111	Positive, subtype ay	Not done	5 (5 probable)	None
Carl et al., USA, 1982 [26]	1979–1980	Retrospective cohort	Obstetrician gynaecologist	740	Positive, subtype ayw	Not done	4 (1 probable, 3 possible) results	HCW noted several episodes of blood on hands after removing gloves

Author, country, publication year	Study period	Study type	Occupation	Persons tested (no.)	Assessment of HBeAg, HBsAg subtype	HBV DNA (copies/mL)	Degree of confirmation	Likely mode of transmission
Couthino et al., The Netherlands, [27]	1979	Case-control	Cardiac surgeon (source A)	540	Not reported, subtype ayw_2	Not done	3 (3 probable)	None
[27] Ditto	Ditto	Case-control	Perfusion technician (source B)	Dto	Positive, subtype adw_2	Not done	11 (8 probable, 3 possible)	Bleeding warts on the technician's hands in 1979
Goodman et al., USA, 1982 [28]	1980	Cross-sectional	Oral surgeon	570	Not done, subtyp adw	Not done	3 (3 probable)	HCW did not wear gloves
Reingold et al., USA, 1982 [29]	1977–1979	Retrospective cohort	Oral surgeon	511	Positive, subtype dy	Not done („strongly positive for HBsAg and HBe")	52 (4 probable, 48 possible)	HCW did not wear gloves and had a generalised eczematous dermatitis
Ahtone et al., USA, 1983 [30]	1980	Cross-sectional[b]	Dentist in private practice	9 (5 staff members; 4 patients)	Positive, no subtyping	Not done	4 possible (no subtyping reported)	HCW did not wear gloves; returned to practice with gloves
Lettau et al., USA, 1986 [31]	1983–1984	Case-control	Obstetrician gynaecologist	683 (274 exposed, 409 control patients)	Positive, subtype aw	Not done	5 (5 probable; 1 additional patient had distinct subtype ayw)	None
Polakoff et al., UK, 1986 [32]	1980–1983	Case series	Perfusion technician	153 (patients with recent operations)	Positive, subtype ay	Not done	6 (6 probable)	HCW did not wear gloves, and had cuts and abrasions on hands
[32] Ditto	1980–1983	Ditto	Surgical registrar	Ditto.	Not reported, subtype ad	Not done	5 (5 probable)	None
Shaw et al., USA,1986 [33]	1984–1985	Retrospective cohort	Dentist	1,133	Positive, subtype ad	Not done	24 (6 probable, 18 possible)	HCW did not wear gloves
Anonymous, UK,1987 [34]	1976–1979	Retrospective cohort	Surgical (gynaecological)	735	Positive, sub-group ay.	Not done (HBsAg "positive to a high titre")	9 (7 probable, 2 possible [anti-HBc positive but HBsAg negative])	None
Welch et al., UK, 1989 [35]	1985–1987	Cross-sectional	Obstetrician-gynaecologist	247	Positive, subtype ad	Not done	22 (6 probable, 16 possible)	None
Anonymous, UK, 1991 [36]	Unknown, publ. in 1991	Case series	Surgeon („surgical colleague")	3	Positive, no subtyping	Not done	3 (3 possible)	None

Author, country, publication year	Study period	Study type	Occupation	Persons tested (no.)	Assessment of HBeAg, HBsAg subtype	HBV DNA (copies/mL)	Degree of confirmation	Likely mode of transmission
Prendergast et al., USA, 1991 [37]	1987	Case series	General surgeon	5 (acute HBV cases)	Positive, subtype ay	Not done	5 (3 probable, 2 possible)	None
Johnston et al., Canada, 1992 [38]	1991	Case series	Orthopaedic surgeon	2	Positive, subtype ay	Not done	2 (1 probable, 1 possible [subtyping not possible])	None
Prentice et al., UK, 1992 [39]	1987–1988	Retrospective cohort	Cardiothoracic surgeon, trainee	280 (of 361 patients at risk)	Positive, subtype ad	Not done	17 (9 probable, 8 possible)	None
Anonymous, UK,1996 [40]	1996	Cross sectional	Member of the operating team (hip surgery)	200 (“approxi-mately”)	Negative	Not done	1 (1 possible)	None
Harpaz et al., USA, 1996 [41]	1991–1992	Retrospective cohort	Cardiothoracic surgeon	187 (144 included in study)	Positive, subtype adw_2	≈$2,5\times10^9$ copies/mL	19 (9 confirmed, 4 probable, 6 possible w/o typing)	None
Halle, UK, 1996 [42], [43]	1996	Case report	Orthopaedic surgeon	1 (230[d])	Negative and HBsAg-positive (precure mutation)	NA (“low concentrations”)	1 (1 confirmed)	None
Heptonstall et al., UK, 1996 [44]	1992–1993	Retrospective cohort study	Cardiothoracic surgeon	310	Positive, subtype adr	Not done	20 (14 confirmed, 2 probable, 4 possible)	None
Mukerjee et al., UK, 1996 [45]	1993	Case-control	General surgeon	16	Positive, subtype adr	Not done	2 (2 confirmed)	None
Incident Investi-gation Team et al., UK, 1997 [46]	1988	Cross-sectional	General surgeon	1	Negative (anti-HBe-positive)	1×10^7 copies/mL	1 (1 confirmed)	None (double-gloving practice unknown)
[46] Ditto.	1988	Ditto	Obstetrician-gynaecologist, trainee	92	Negative (anti-HBe-positive)	4.4×10^6 copies/mL	3 (3 confirmed)	None (double-gloving practice only occasionally)
[46] Ditto	1988	Ditto	Obstetrician-gynaecologist, trainee	111	Negative (anti-HBe-negative)	5.5×10^6 copies/mL	1 (1 confirmed)	None (double-gloving practice only occasionally)
[46] Ditto	1988	Ditto	General sur-geon, urolo-gist, clinical assistant	21	Negative (anti-HBe-positive)	2.5×10^5 copies/mL	1 (1 confirmed)	None (double-gloving practice only occasionally)
Sundkvist et al., UK, 1996 [47]	1996	Cross-sectional	Orthopaedic surgeon	189	Negative (anti-HBe-positive)	Not done	1 (1 confirmed)	None

Author, country, publication year	Study period	Study type	Occupation	Persons tested (no.)	Assessment of HBeAg, HBsAg subtype	HBV DNA (copies/mL)	Degree of confirmation	Likely mode of transmission
Oliver et al., UK, 1999 [48]	1993–1994	Retrospective cohort	General surgeon, trainee	390[e]	Positive, no subtyping	Not done	11 (1 confirmed, 10 possible)	None
[48] Ditto	1994	Ditto	General surgeon, trainee	96	Positive, no subtyping	Not done	2 (2 possible)	None
[48] Ditto	1994	Ditto	Urologist, trainee	28	Positive, no subtyping	Not done	1 (1 possible)	None
Walsh et al., UK, 1999 [49]	1990–1992	Retrospective cohort	Acupuncture practitioner	360 (39% of all patients identified)	Positive, subtype adw2	Not done	5 (2 confirmed by PCR-SSCPA [Polymerase Chain Reaction – Single-Strand Conformation Polymorphism Analysis, 3 possible])	None
Hepatitis B Outbreak Investigation Team, Canada, 2000 [50]	1991–1996	Retrospective cohort	Electroencephalogram technician	9,620	Positive, no subtyping	Not done (HBV DNA patterns were identical, but copies/mL were not determined)	75 (4 confirmed, 71 possible)	HCW did not wear gloves and used reusable subdermal EEG electrodes
Molynaux et al., UK, 2000 [51]	1999	Cross-sectional	Cardiothoracic surgeon	123	Negative (anti-HBe-positive)	$1.03×10^6$ copies/mL	2 (2 confirmed)	None
Spikermann et al., The Netherlands, 2002 [52],	1995–1999	Retrospective cohort	General surgeon	1,564	Positive, subtype adw2	$5.0×10^9$ HBV copies/mL	27 (7* confirmed, 20 possible)[f] ; 1 secondary transmission to a patient´s wife excluded.	HCW noted glove perforations
Cordon et al., UK, 2003 [53]	1988–1997	Cross-sectional	Surgeons (n=8)	8	Negative (all 8 surgeons)	$4×10^4$–$1,5×10^9$ copies/mL (Roche assay values)	8 (confirmed). All surgeons carried codon 28 variants and transmitted these variants to their patients	None
Poujol et al., France, 2005 [54]	2005	Case-contact tracing	Anaesthetic nurse (vascular surgery)	28 (6 patients, 22 HCW)	Not reported (HBsAg-positive)	Not done	1 (1confirmed by molecular analysis of HBV gene S and C)	HCW did not wear gloves and reported needle sticks on several occasions
Smellie et al., UK, 2006 [55]	1998	Case-contact tracing	Surgical house officer	3,381 (231 staff members and 3,150 potentially treated patients)	Positive, no subtyping	Not done	2 (2 confirmed)	When performing venepuncture or inserting intravenous lines, the HCW did not wear gloves.

Author, country, publication year	Study period	Study type	Occupation	Persons tested (no.)	Assessment of HBeAg, HBsAg subtype	HBV DNA (copies/mL)	Degree of confirmation	Likely mode of transmission
Laurenson et al., UK, 2007 [56]	2001	Case series	General surgeon	3	Negative, subtype ayw2	>10^6 copies/mL	3 (3 confirmed)	None
Enfield et al., USA, 2013 [57]	2009	Retrospective cohort	Orthopaedic surgeon	232	Positive, subtype ayw3	≈9.42×10^7 copies/mL (17.9 million IU/mL)[g]	8 (2 confirmed, 6 possible)	None
Sugimoto et al., Japan, 2013 [58]	2010	Case-contact tracing	Obstetrician-gynaecologist	63	Positive, no subtyping	1.6×10^9 copies/mL	1 (1 confirmed)	None

[a] No subtyping done in 1 HBsAg-positive patient and 3 patients were HBsAg-negative.

[b] Patients with clinical HBV infection treated in the preceding 3 years by the dentist were found by comparing a list of HBV cases reported to the county in the same time period.

[c] HBV DNA concentration of 15 ng/mL corresponds to approximately 2.5×10^9 copies/mL.

[d] A total of 230 patients were checked for HBV markers but the results were not reported.

[e] 514 patients were asked by questionnaire but only those who had previously contracted acute hepatitis B were serologically tested.

[f] Although 55 patients were stated “associated”, only 42 patients had been tested for HBsAg, of those were 33 positive. Subtyping of HBsAg-positive samples was only available for 11 patients; of whose 1 HBsAg-positive patient with subtype ad differed from the surgeon´s subtype.

[g] For the VERSANT HBV bDNA 3.0 Assay used in this study, a study reports a conversion factor of 1 IU/mL≈5.26 copies/mL. Therefore, a viral load of 17.9 million IU/mL corresponds to approximately 9.42×10^7 copies/mL.

References

1. Robert Koch-Institut. Schutzimpfung gegen Hepatitis B: Häufig gestellte Fragen und Ant-worten. Berlin: RKI; 2024 Nov 21. Available from: https://www.rki.de/SharedDocs/FAQs/DE/Impfen/HepatitisB/FAQ-Liste_HepB_Impfen.html#entry_16870840
2. Singh J, Stoitsova S, Zakrzewska K, Henszel L, Rosińska M, Duffell E. Healthcare-associated hepatitis B and C transmission to patients in the EU/EEA and UK: a systematic review of reported outbreaks between 2006 and 2021. BMC Public Health. 2022 Dec;22(1):2260. DOI: 10.1186/s12889-022-14726-0
3. Robert Koch-Institut. Ratgeber Infektionskrankheiten 17. Folge: Hepatitis B. Epid Bull. 2000 Aug 18;33:263-7.
4. Brodzinski A, Neumeyer-Gromen A, Dudareva S, Zimmermann R, Latza U, Bremer V, Poethko-Müller C. Hepatitis-B-Virus-Infektionen und impfinduzierte Immunität: die Rolle von soziodemografischen Determinanten: Ergebnisse der „Studie zur Gesundheit Erwachsener in Deutschland" (DEGS1, 2008–2011) [Hepatitis B virus infection and vaccine-induced immunity: the role of sociodemographic determinants: Results of the study "German Health Interview and Examination Survey for Adults" (DEGS1, 2008-2011)]. Bundesgesundheitsblatt Gesundheitsforschung Gesundheitsschutz. 2022 Feb;65(2):159-169. DOI: 10.1007/s00103-021-03473-z
5. Heintges T, Häussinger D. Hepatitis B: Infektion - Therapie - Prophylaxe. Stuttgart: Thieme; 2006. DOI: 10.1055/b-002-43886
6. Dulon M, Stranzinger J, Wendeler D, Nienhaus A. Berufsbedingte Infektionskrankheiten bei Beschäftigten im Gesundheitsdienst 2023. Zentralblatt für Arbeitsmedizin, Arbeitsschutz und Ergonomie. 2025. DOI: 10.1007/s40664-025-00579-y
7. Lettau LA, Smith JD, Williams D, Lundquist WD, Cruz F, Sikes RK, Hadler SC. Transmission of hepatitis B with resultant restriction of surgical practice. JAMA. 1986 Feb;255(7):934-7.
8. Perry JL, Pearson RD, Jagger J. Infected health care workers and patient safety: a double standard. Am J Infect Control. 2006 Jun;34(5):313-9. DOI: 10.1016/j.ajic.2006.01.004
9. Carlson AL, Perl TM. Health care workers as source of hepatitis B and C virus transmission. Clin Liver Dis. 2010 Feb;14(1):153-68; x. DOI: 10.1016/j.cld.2009.11.003
10. Gerlich WH. Hepatitis B und CUbertragungsgefahr auf Patienten durch infiziertes medizinisches Personal [Hepatitis B and C. Risk of transmission from infected health care workers to patients]. Bundesgesundheitsblatt Gesundheitsforschung Gesundheitsschutz. 2004 Apr;47(4):369-78. DOI: 10.1007/s00103-004-0811-x
11. Lewis JD, Enfield KB, Sifri CD. Hepatitis B in healthcare workers: Transmission events and guidance for management. World J Hepatol. 2015 Mar;7(3):488-97. DOI: 10.4254/wjh.v7.i3.488
12. Page MJ, McKenzie JE, Bossuyt PM, Boutron I, Hoffmann TC, Mulrow CD, Shamseer L, Tetzlaff JM, Akl EA, Brennan SE, Chou R, Glanville J, Grimshaw JM, Hróbjartsson A, Lalu MM, Li T, Loder EW, Mayo-Wilson E, McDonald S, McGuinness LA, Stewart LA, Thomas J, Tricco AC, Welch VA, Whiting P, Moher D. The PRISMA 2020 statement: an updated guideline for reporting systematic reviews. BMJ. 2021 Mar;372:n71. DOI: 10.1136/bmj.n71

13. Ronsin C, Pillet A, Bali C, Denoyel GA. Evaluation of the COBAS AmpliPrep-total nucleic acid isolation-COBAS TaqMan hepatitis B virus (HBV) quantitative test and comparison to the VERSANT HBV DNA 3.0 assay. J Clin Microbiol. 2006 Apr;44(4):1390-9. DOI: 10.1128/JCM.44.4.1390-1399.2006

14. Munn Z, Moola S, Lisy K, Riitano D, Tufanaru C. Methodological guidance for systematic reviews of observational epidemiological studies reporting prevalence and cumulative incidence data. Int J Evid Based Healthc. 2015 Sep;13(3):147-53. DOI: 10.1097/XEB.0000000000000054

15. The Joanna Briggs Institute. The Joanna Briggs Institute Critical Appraisal tools for use in JBI Systematic Reviews: Checklist for Prevalence Studies. 2017. Available from: https://jbi.global/critical-appraisal-tools

16. Goodwin D, Fannin SL, McCracken BB. An oral-surgeon related hepatitis B outbreak. Calif Morbid. 1976 Apr 16;(14).

17. Garibaldi RA, Hatch FE, Bisno AL, Hatch MH, Gregg MB. Nonparenteral serum hepatitis. Report of an outbreak. JAMA. 1972 May;220(7):963-6.

18. Grob PJ, Moeschlin P. Letter: Risk to contacts of a medical practitioner carrying HBs AG. N Engl J Med. 1975 Jul;293(4):197. DOI: 10.1056/NEJM197507242930412

19. Levin ML, Maddrey WC, Wands JR, Mendeloff AL. Hepatitis B transmission by dentists. JAMA. 1974 May;228(9):1139-40.

20. Snydman DR, Hindman SH, Wineland MD, Bryan JA, Maynard JE. Nosocomial viral hepatitis B. A cluster among staff with subsequent transmission to patients. Ann Intern Med. 1976 Nov;85(5):573-7. DOI: 10.7326/0003-4819-85-5-573

21. Rimland D, Parkin WE, Miller GB Jr, Schrack WD. Hepatitis B outbreak traced to an oral surgeon. N Engl J Med. 1977 Apr;296(17):953-8. DOI: 10.1056/NEJM197704282961701

22. Acute hepatitis B associated with gynaecological surgery. Lancet. 1980 Jan 5;1(8158):1-6. DOI: 10.1016/S0140-6736(80)90548-6

23. Grob PJ, Bischof B, Naeff F. Cluster of hepatitis B transmitted by a physician. Lancet. 1981 Nov;2(8257):1218-20. DOI: 10.1016/s0140-6736(81)91450-1

24. Hadler SC, Sorley DL, Acree KH, Webster HM, Schable CA, Francis DP, Maynard JE. An outbreak of hepatitis B in a dental practice. Ann Intern Med. 1981 Aug;95(2):133-8. DOI: 10.7326/0003-4819-95-2-133

25. Haerem JW, Siebke JC, Ulstrup J, Geiran O, Helle I. HBsAG transmission from a cardiac surgeon incubating hepatitis B resulting in chronic antigenemia in four patients. Acta Med Scand. 1981;210(5):389-92. DOI: 10.1111/j.0954-6820.1981.tb09836.x

26. Carl M, Blakey DL, Francis DP, Maynard JE. Interruption of hepatitis B transmission by modification of a gynaecologist's surgical technique. Lancet. 1982 Mar;1(8274):731-3. DOI: 10.1016/s0140-6736(82)92636-8

27. Coutinho RA, Albrecht-van Lent P, Stoutjesdijk L, Meerburg-Snarenberg P, Couroucé-Pauty AM, van Dijk BA, Kloek J. Hepatitis B from doctors. Lancet. 1982 Feb;1(8267):345-6. DOI: 10.1016/s0140-6736(82)91607-5

28. Goodman RA, Ahtone JL, Finton RJ. Hepatitis B transmission from dental personnel to patients: unfinished business. Ann Intern Med. 1982 Jan;96(1):119. DOI: 10.7326/0003-4819-96-1-119_1

29. Reingold AL, Kane MA, Murphy BL, Checko P, Francis DP, Maynard JE. Transmission of hepatitis B by an oral surgeon. J Infect Dis. 1982 Feb;145(2):262-8. DOI: 10.1093/infdis/145.2.262

30. Ahtone J, Goodman RA. Hepatitis B and dental personnel: transmission to patients and prevention issues. J Am Dent Assoc. 1983 Feb;106(2):219-22. DOI: 10.14219/jada.archive.1983.0416

31. Lettau LA, Smith JD, Williams D, Lundquist WD, Cruz F, Sikes RK, Hadler SC. Transmission of hepatitis B with resultant restriction of surgical practice. JAMA. 1986 Feb;255(7):934-7.

32. Polakoff S. Acute hepatitis B in patients in Britain related to previous operations and dental treatment. Br Med J (Clin Res Ed). 1986 Jul;293(6538):33-6. DOI: 10.1136/bmj.293.6538.33

33. Shaw FE Jr, Barrett CL, Hamm R, Peare RB, Coleman PJ, Hadler SC, Fields HA, Maynard JE. Lethal outbreak of hepatitis B in a dental practice. JAMA. 1986 Jun;255(23):3260-4.

34. Acute hepatitis B following gynaecological surgery: A district control of infection officer. Journal of Hospital Infection. 1987; 9(1):34-8. DOI: 10.1016/0195-6701(87)90092-2

35. Welch J, Webster M, Tilzey AJ, Noah ND, Banatvala JE. Hepatitis B infections after gynaecological surgery. Lancet. 1989 Jan;1(8631):205-7. DOI: 10.1016/s0140-6736(89)91213-0

36. Surgeons who are hepatitis B carriers. BMJ. 1991 Jul 20;303(6795):184-5.

37. Prendergast TJ Jr, Teitelbaum S, Peck B. Transmission of hepatitis B by a surgeon. West J Med. 1991 Mar;154(3):353.

38. Johnston BL, MacDonald S, Lee S, LeBlanc JC, Gross M, Schlech WF, Chaudhary R, Langille D. Nosocomial hepatitis B associated with orthopedic surgery--Nova Scotia. Can Commun Dis Rep. 1992 Jun 26;18(12):89-90.

39. Prentice MB, Flower AJ, Morgan GM, Nicholson KG, Rana B, Firmin RK, Mitchell CJ. Infection with hepatitis B virus after open heart surgery. BMJ. 1992 Mar;304(6829):761-4. DOI: 10.1136/bmj.304.6829.761

40. Possible transmission of hepatitis B virus from a health care worker to a patient. Commun Dis Rep CDR Wkly. 1996 Aug 16;6(33):283, 286.

41. Harpaz R, Von Seidlein L, Averhoff FM, Tormey MP, Sinha SD, Kotsopoulou K, Lambert SB, Robertson BH, Cherry JD, Shapiro CN. Transmission of hepatitis B virus to multiple patients from a surgeon without evidence of inadequate infection control. N Engl J Med. 1996 Feb;334(9):549-54. DOI: 10.1056/NEJM199602293340901

42. Halle M. Patients want ban on operations by doctors with hepatitis B. BMJ. 1996 Sep;313(7057):576. DOI: 10.1136/bmj.313.7057.576a

43. Halle M. Surgeon had mutant form of hepatitis B. BMJ. 1996 Sep;313(7060):771. DOI: 10.1136/bmj.313.7060.771a

44. Heptonstall J. Lessons from two linked clusters of acute hepatitis B in cardiothoracic surgery patients. Commun Dis Rep CDR Rev. 1996 Aug 16;6(9):R119-25.

45. Mukerjee AK, Westmoreland D, Rees HG. Response to the discovery of two practising surgeons infected with hepatitis B. Commun Dis Rep CDR Rev. 1996 Aug 16;6(9):R126-8.

46. Incident Investigation Teams and Others. Transmission of hepatitis B to patients from four infected surgeons without hepatitis B e antigen. N Engl J Med. 1997 Jan;336(3):178-84. DOI: 10.1056/NEJM199701163360304

47. Sundkvist T, Hamilton GR, Rimmer D, Evans BG, Teo CG. Fatal outcome of transmission of hepatitis B from an e antigen negative surgeon. Commun Dis Public Health. 1998 Mar;1(1):48-50.

48. Oliver SE, Woodhouse J, Hollyoak V. Lessons from patient notification exercises following the identification of hepatitis B e antigen positive surgeons in an English health region. Commun Dis Public Health. 1999 Jun;2(2):130-6.

49. Walsh B, Maguire H, Carrington D. Outbreak of hepatitis B in an acupuncture clinic. Commun Dis Public Health. 1999 Jun;2(2):137-40.

50. An outbreak of hepatitis B associated with reusable subdermal electroencephalogram electrodes. Hepatitis B Outbreak Investigation Team. CMAJ. 2000 Apr 18;162(8):1127-31.

51. Molyneaux P, Reid TM, Collacott I, McIntyre PG, Dillon JF, Laing RB. Acute hepatitis B in two patients transmitted from an e antigen negative cardiothoracic surgeon. Commun Dis Public Health. 2000 Dec;3(4):250-2.

52. Spijkerman IJ, van Doorn LJ, Janssen MH, Wijkmans CJ, Bilkert-Mooiman MA, Coutinho RA, Weers-Pothoff G. Transmission of hepatitis B virus from a surgeon to his patients during high-risk and low-risk surgical procedures during 4 years. Infect Control Hosp Epidemiol. 2002 Jun;23(6):306-12. DOI: 10.1086/502056

53. Corden S, Ballard AL, Ijaz S, Barbara JA, Gilbert N, Gilson RJ, Boxall EH, Tedder RS. HBV DNA levels and transmission of hepatitis B by health care workers. J Clin Virol. 2003 May;27(1):52-8. DOI: 10.1016/s1386-6532(02)00127-0

54. Poujol I, Floret N, Servant-Delmas A, Marquant A, Laperche S, Antona D, Lot F, Coignard B. Hepatitis B virus transmission from a nurse to a patient, France, 2005. Euro Surveill. 2008 May;13(21):. DOI: 10.2807/ese.13.21.18877-en

55. Smellie MK, Carman WF, Elder S, Walker D, Lobidel D, Hardie R, Downie G, McMenamin J, Cameron S, Morrison D, Armstrong J, Goldberg D. Hospital transmission of hepatitis B virus in the absence of exposure prone procedures. Epidemiol Infect. 2006 Apr;134(2):259-63. DOI: 10.1017/S0950268805004942

56. Laurenson IF, Jones DG, Hallam NF, Saunders CJ, Fraser DM, Carman WF. Transmission of hepatitis B virus from a vaccinated healthcare worker. J Hosp Infect. 2007 Aug;66(4):393-4. DOI: 10.1016/j.jhin.2007.05.001

57. Enfield KB, Sharapov U, Hall KK, Leiner J, Berg CL, Xia GL, Thompson ND, Ganova-Raeva L, Sifri CD. Transmission of hepatitis B virus from an orthopedic surgeon with a high viral load. Clin Infect Dis. 2013 Jan;56(2):218-24. DOI: 10.1093/cid/cis869

58. Sugimoto S, Nagakubo S, Ito T, Tsunoda Y, Imamura S, Tamura T, Morohoshi Y, Koike Y, Fujita Y, Ito S, Fujita S, Tachikawa N, Komatsu H. A case of acute hepatitis B related to previous gynecological surgery in Japan. J Infect Chemother. 2013 Jun;19(3):524-9. DOI: 10.1007/s10156-012-0477-5

59. Recommendations for preventing transmission of human immunodeficiency virus and hepatitis B virus to patients during exposure-prone invasive procedures. MMWR Recomm Rep. 1991 Jul 12;40(RR-8):1-9.

60. Chen CJ, Yang HI, Iloeje UH; REVEAL-HBV Study Group. Hepatitis B virus DNA levels and outcomes in chronic hepatitis B. Hepatology. 2009 May;49(5 Suppl):S72-84. DOI: 10.1002/hep.22884

61. Tedder RS, Ijaz S, Gilbert N, Barbara JA, Corden SA, Gilson RJ, Boxall EH. Evidence for a dynamic host-parasite relationship in e-negative hepatitis B carriers. J Med Virol. 2002 Dec;68(4):505-12. DOI: 10.1002/jmv.10241

62. Gunson RN, Shouval D, Roggendorf M, Zaaijer H, Nicholas H, Holzmann H, de Schryver A, Reynders D, Connell J, Gerlich WH, Marinho RT, Tsantoulas D, Rigopoulou E, Rosenheim M, Valla D, Puro V, Struwe J, Tedder R, Aitken C, Alter M, Schalm SW, Carman WF; European Consensus Group. Hepatitis B virus (HBV) and hepatitis C virus (HCV) infections in health care workers (HCWs): guidelines for prevention of transmission of HBV and HCV from HCW to patients. J Clin Virol. 2003 Aug;27(3):213-30. DOI: 10.1016/s1386-6532(03)00087-8

63. Glebe D, van Bömmel F, Dudareva S, Gärtner B, Monazahian M, Roß S, Rösler J, Slanina H, Spickhoff A, Thanheiser M, Schüttler CG. Prävention der nosokomialen Übertragung von Hepatitis-B-Virus (HBV) und Hepatitis-C-Virus (HCV) durch im Gesundheitswesen Tätige: Empfehlungen der Deutschen Vereinigung zur Bekämpfung der Viruskrankheiten (DVV) e.V. Bundesgesundheitsblatt Gesundheitsforschung Gesundheitsschutz. 2020 Feb;63(2):218-225. DOI: 10.1007/s00103-019-03084-9

64. Cornberg M, Sandmann L, Protzer U, Niederau C, Tacke F, Berg T, Glebe D, Jilg W, Wedemeyer H, Wirth S, Höner Zu Siederdissen C, Lynen-Jansen P, van Leeuwen P, Petersen J; Collaborators:. S3-Leitlinie der Deutschen Gesellschaft für Gastroenterologie, Verdauungs- und Stoffwechselkrankheiten (DGVS) zur Prophylaxe, Diagnostik und Therapie der Hepatitis-B-Virusinfektion – (AWMF-Register-Nr. 021-11). Z Gastroenterol. 2021 Jul;59(7):691-776. DOI: 10.1055/a-1498-2512

65. Department of Health. Hepatitis B infected health care workers: guidance on implementation of Health Service Circular 2000/020 of the Department of Health. London: Department of Health; 2000. p. 1–11. Available from: https://webarchive.nationalarchives.gov.uk/ukgwa/20120907233941/http://www.dh.gov.uk/en/Publicationsandstatistics/Publications/PublicationsPolicyAndGuidance/DH_4008156

66. Department of Health. Hepatitis B infected healthcare workers and antiviral therapy: best practice guidance of the Department of Health. London: Department of Health; 2007. p. 1–16. Available from: https://www.gov.uk/government/groups/uk-advisory-panel-for-healthcare-workers-infected-with-bloodborne-viruses

67. Desai M, Hibber M, Njoroge J; UK Advisory Panel for Healthcare Workers Infected with Bloodborne Viruses (UKAP). Integrated guidance on health clearance of healthcare workers and the management of healthcare workers living with blood-borne viruses (hepatitis B, hepatitis C and HIV). London: UKAP; 2024 Apr.

68. Commissie Preventie Iatrogene Transmissie van HBV, HCV en HIV. Landelijke richtlijn preventie transmissie van hepatitis van medisch personeel naar patiënten. 3e ed. Bilthoven: Rijksinstituut voor Volksgezondheid en Milieu (RIVM); 2012 Sep 10. Available from: https://lci.rivm.nl/richtlijnen/hepatitis-c/landelijke-richtlijn-preventie-transmissie-van-hepatitis-b-van-medisch-personeel-naar

69. Commissie Preventie Iatrogene Transmissie van HBV, HCV en HIV. Landelijke richtlijn preventie transmissie van hepatitis B van medisch personeel naar patiënten. 4e ed. Bilthoven: Rijksinstituut voor Volksgezondheid en Milieu (RIVM); 2021 Jul. Available from: https://lci.rivm.nl/overig/preventie-iatrogene-transmissie-hepatitis-b

70. Henderson DK, Dembry LM, Sifri CD, Palmore TN, Dellinger EP, Yokoe DS, Grady C, Heller T, Weber D, Del Rio C, Fishman NO, Deloney VM, Lundstrom T, Babcock HM. Management of healthcare personnel living with hepatitis B, hepatitis C, or human immunodeficiency virus in US healthcare institutions. Infect Control Hosp Epidemiol. 2022 Feb;43(2):147-155. DOI: 10.1017/ice.2020.458

71. Henderson DK, Dembry L, Fishman NO, Grady C, Lundstrom T, Palmore TN, Sepkowitz KA, Weber DJ; Society for Healthcare Epidemiology of America. SHEA guideline for management of healthcare workers who are infected with hepatitis B virus, hepatitis C virus, and/or human immunodeficiency virus. Infect Control Hosp Epidemiol. 2010 Mar;31(3):203-32. DOI: 10.1086/650298

72. Centers for Disease Control and Prevention (CDC). Updated CDC recommendations for the management of hepatitis B virus-infected health-care providers and students. MMWR Recomm Rep. 2012 Jul 6;61(RR-3):1-12.
